# Asthma, COPD & ACOS: -Omics Approach to Refine Treatable Traits in These Conditions

**Published:** 2017

**Authors:** Kian Fan CHUNG

**Affiliations:** National Heart & Lung Institute, Imperial College, London UK

Overlapping characteristics of asthma and COPD frequently coexist in the same patient and the term ACOS has been applied to these patients. Patients with both fixed airflow obstruction and bronchodilator reversibility or bronchial hyperresponsiveness in addition to those with eosinophilic inflammation, a history of allergic disease, and cigarette smoke exposure have been considered to be part of ACOS. In ECLIPSE, symptoms and exacerbations were higher among ACOS patients than COPD patients, irrespective of their severity of airflow limitation, age, sex, lung function and exacerbation history. In addition, ACOS has been associated with larger number of hospital admissions, and use of health-care resources. Clinical phenotypes of ACOS have been divided into those with asthma (asthma-ACOS) or COPD (COPD-ACOS) prominence.

The focus on ACOS has raised the issue as to whether asthma and COPD have similar origins. ACOS is not a disease entity, just as asthma and COPD are not, being defined by a set of symptoms or changes in lung function. There is a pressing need for a definition based on phenotyping and endotyping rather than on clinical description alone, as is it is the case for asthma and COPD. In this context, instead of ACOS, it has been proposed to identify “treatable traits” such as airflow obstruction, exacerbations and eosinophilic inflammation based on understanding of the mechanistic pathways. Both asthma and COPD together with ACOS share these treatable traits.

Molecular phenotyping of UBIOPRED severe asthma cohort using sputum has identified one Th2-high containing phenotype with two Th2-low endotypes driven by inflammasome activation and mitochondrial oxidative stress, respectively. We have applied these molecular signatures found in UBIOPRED severe asthma cohort to the GLUCOLD cohort and found that there was a Th2-high signature in a cluster where treatment with inhaled corticosteroids reduced the Th2-signature. Non-Th2 clusters included those with inflammasome component and mitochondrial oxidative stress with ageing. Analysis of the smoking severe asthma cohort showed that smoking asthmatics form a distinct part of asthma-ACOS. Defining the endotypes of treatable traits will be a better path towards precision medicine of these 3 diseases.

